# Evolution at Two Levels: On Genes and Form

**DOI:** 10.1371/journal.pbio.0030245

**Published:** 2005-07-12

**Authors:** Sean B Carroll

## Abstract

Emerging knowledge about organismal evolution suggests that changes in the regulation of gene expression have played a major role - a thesis proposed 30 years ago by King and Wilson.

In their classic paper “Evolution at Two Levels in Humans and Chimpanzees,” published exactly 30 years ago, Mary-Claire King and Allan Wilson described the great similarity between many proteins of chimpanzees and humans [1]. They concluded that the small degree of molecular divergence observed could not account for the anatomical or behavioral differences between chimps and humans. Rather, they proposed that evolutionary changes in anatomy and way of life are more often based on changes in the mechanisms controlling the expression of genes than on sequence changes in proteins.

This article was a milestone in three respects. First, because it was the first comparison of a large set of proteins between closely related species, it may be considered one of the first contributions to “comparative genomics” (although no such discipline existed for another two decades). Second, because it extrapolated from molecular data to make inferences about the evolution of form, it may also be considered a pioneering study in evolutionary developmental biology. And third, its focus on the question of human evolution and human capabilities, relative to our closest living relative, marked the beginning of the quest to understand the genetic basis of the origins of human traits. Like much of Wilson and his colleagues' body of work, this contribution had a great influence on paleoanthropologists as well as molecular biologists.

The 30th anniversary of this landmark article arrives at a moment when comparative genomics, evolutionary developmental biology, and evolutionary genetics are pouring forth unprecedented amounts of new data, and the entire chimpanzee genome is available for study. It is therefore an opportune time to examine what has been and is being revealed about the relationship between evolution at the two levels of molecules and organisms, and to assess the status of King and Wilson's hypothesis concerning the predominant role of regulatory mutations in organismal evolution.

King and Wilson used the phrase “ways of life” to include both physiology and behavior (M.-C. King, personal communication) and proposed that the evolution of both anatomy and ways of life was governed by regulatory changes in the expression of genes. From the outset of this review, I make the sharp distinction between the evolution of anatomy and the evolution of physiology. Changing the size, shape, number, or color patterns of physical traits is fundamentally different from changing the chemistry of physiological processes. There is ample evidence from studies of the evolution of proteins directly involved in animal vision [2], respiration [3], digestive metabolism [4], and host defense [5] that the evolution of coding sequences plays a key role in some (but not all) important physiological differences between species. In contrast, the relative contribution of coding or regulatory sequence evolution to the evolution of anatomy stands as the more open question, and will be my primary focus.

The amount of direct evidence currently in hand is modest, and includes examples of both the evolution of coding and of non-coding, regulatory sequences contributing to morphological evolution. However, I will develop the argument, on the basis of theoretical considerations and a rapidly expanding body of empirical studies, that regulatory sequence evolution must be the major contributor to the evolution of form.

This conclusion poses particular challenges to comparative genomics. While we are often able to infer coding sequence function from primary sequences, we are generally unable to decipher functional properties from mere inspection of non-coding sequences. This has led to a bias in comparative genomics and evolutionary genetics toward the analysis and reporting of readily detectable events in coding regions, such as gene duplications and protein sequence evolution, while non-coding, regulatory sequences are often ignored. However, approximately two-thirds of all sequences under purifying selection in our genome are non-coding [6]. One consequence of the underconsideration of non-coding, regulatory sequences is unrealistic expectations about what can currently be learned about the genetic bases of morphological diversity from comparisons of genome sequences alone. The visible diversity of any group is not reflected by the most visible components of gene diversity—that is, the diversity of gene number or of coding sequences. In order to understand the evolution of anatomy, we have to study and understand regulatory sequences, as well as the proteins that connect them into the regulatory circuits that govern development. I will begin with some historical and theoretical considerations about regulatory and coding sequence evolution, then delve into the insights offered by specific experimental models of anatomical evolution, and finally, I will revisit King and Wilson's original focus and discuss how our emerging knowledge of the evolution of form bears on current efforts to understand human evolution.

## A Brief History of Regulatory Thinking

Almost 50 years ago, as the first sequences of various proteins from different species were determined, the potential significance of macromolecules for understanding evolutionary processes was quickly recognized [7]. The great similarity among homologous proteins of different species was noted early [8] and raised the question to what degree such sequence changes were functionally significant [9]. With the advent of the operon model of gene regulation [10], some biologists such as Emile Zuckerkandl began to consider the possible role of “controller genes” in evolution, including in the origin of humans from ape ancestors [11]. One of the most widely noted series of theoretical contributions in this period was Roy Britten and Eric Davidson's models for gene regulation in higher organisms, which had an explicit emphasis on the importance of gene regulation in evolution [12,13].

The most influential single publication of this era, however, was Susumu Ohno's book *Evolution by Gene Duplication* [14]. Ohno focused on the importance of gene redundancy in allowing “forbidden” mutations to occur that could impart new functions to proteins. His opening motto, “natural selection merely modified, while redundancy created,” reflected a view of natural selection as a largely purifying, conservative process. Ohno insisted that “allelic mutations of already existing gene loci cannot account for major changes in evolution.” He proposed that the duplication of regulatory genes and their control regions must have contributed greatly to the evolution of vertebrates. But the book focused exclusively on the evolution of new proteins and did not consider the creative potential of non-coding, regulatory sequences in evolutionary diversification (see [15]).

It was against this backdrop that Allan Wilson and his colleagues began a series of investigations into the relationship between chromosomal evolution, protein evolution, and anatomical evolution in birds [16], mammals [17], frogs [18], and apes [1]. In each of four studies, the discrepancy between the evolution of proteins and the evolution of anatomy led to the conclusion that evolutionary changes in “regulatory systems” were responsible for the evolution of anatomy. Francois Jacob similarly suggested that divergence and specialization result from mutations altering “regulatory circuits” rather than chemical structures [19].

The relative contributions of different mechanisms to the evolution of anatomy depend upon both what is genetically possible, and what is permitted by natural selection. Before I delve into the data directly concerning the evolution of anatomy, and how well it fulfills King and Wilson's original expectations, it will be valuable to consider what mechanisms are available and what parameters will govern their utilization in evolution, in light of what we now understand about how genes function in multicellular organisms.

## Pleiotropy and the Genetic Architecture of Multicellular Organisms

One critical parameter that affects the relative contribution of different genetic mechanisms to anatomical variation is the pleiotropy of mutations [20]. In general, it is expected that mutations with greater pleiotropic effects will have more deleterious effects on organismal fitness and will be a less common source of variation in form than mutations with less widespread effects.

Over the past 30 years, several key features of gene structure, function, and regulation in multicellular organisms have emerged that govern the pleiotropy of mutations and thus shape the capacity of species to generate anatomical variation and to evolve (see Box 1). Because of these features, mutations in different genes and different parts of genes (that is, non-coding and coding sequences) can differ dramatically in their degree of pleiotropy. For example, a mutation in the coding region of a transcription factor that functions in multiple tissues may directly affect all of the genes the protein regulates. In contrast, a mutation in a single cis-regulatory element will affect gene expression only in the domain governed by that element.

**Box 1. Key Genetic Features of Multicellular Organisms**

**Individual regulatory proteins function in many different contexts.** Signaling proteins, their receptors, signal transducers, and most transcription factors are deployed in multiple tissues, organs, or body parts. The function of each regulatory protein is context-dependent, with different genetic targets and morphogenetic outcomes in different tissues.

**The expression of individual genes is regulated by multiple, modular *cis*-regulatory elements.** The tissue-specific and temporal control of gene expression, particularly of genes encoding the regulatory proteins that shape pattern formation and cell differentiation in animals, is typically governed by arrays of discrete regulatory elements embedded in regions that flank coding regions and lie within introns [23].

**Many regulatory proteins are members of large families and can overlap in function.** More than 20% of human genes and a much larger fraction of plant genes belong to families [75] that are the product of the duplication and evolutionary divergence of ancestral genes.

**Multiple protein forms may be encoded by single genetic loci.** Through the use of alternative promoters and RNA splice sites, multiple mRNAs encoding different protein products are often produced from a single locus. Alternative protein forms (isoforms) may function in different contexts and/or possess different activities.

John Gerhart and Marc Kirschner [21,22] have discussed in depth how certain features of animal genetic regulatory systems influence “evolvability”—the capacity to generate tolerable, heritable variation. For instance, redundancy reduces constraint on change by circumventing or minimizing the potentially deleterious effects of some mutations. Compartmentation also facilitates change; by uncoupling variation in one process from variation in another, pleiotropy is decreased.

**Figure 1:**
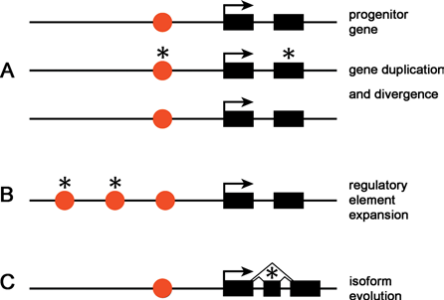
Different Modes of Gene Evolution Increase the Diversity of Gene Function and Minimize Pleiotropy The function of a progenitor gene with the simple structure of one *cis*-regulatory element (red circle) and a pair of exons (black rectangles) can be expanded and diversified in several ways. (A) Gene duplication followed by mutations (asterisks) in either coding or regulatory sequences of the initially identical paralogs will produce genes that may be expressed in different ways or proteins with distinct functions, while the original function can be maintained. (B) An expansion in the number of *cis*-regulatory elements by any of a number of means (transposition, rearrangement, duplication, point mutation) can expand the number of tissues in which the gene is active, but preserves the original function. (C) The evolution of a new exon and splicing sites creates the potential for alternative forms of a protein to be made. Mutations in alternative exons (asterisks) need not affect the original function of the protein.

Several genetic features contribute to redundancy and compartmentation. For example, gene duplication creates initially redundant paralogs. Mutations that may have been deleterious in the ancestral gene may be tolerated and allow for the “exploration” of new variation, which can occur in coding or regulatory sequences, or both (Figure 1A). Likewise, the expanded number and diversity of cis-regulatory elements establishes compartmentation by enabling the independent control of gene transcription in different body parts (Figure 1B). The use of alternative promoters and RNA splice sites also contributes to compartmentation by enabling tissue- or cell-typespecific production of alternative forms of a protein (Figure 1C). Variation may arise either in regulatory sequences governing promoter use or splice site choice, or in coding sequences of exons. The three mechanisms gene duplication, regulatory sequence expansion and diversification, and alternative protein isoform expression accomplish essentially the same general result—they increase the sources of variation and minimize the pleiotropy associated with the evolution of coding sequences. The global question of the genetic basis of the evolution of form then boils down to the relative contribution of gene duplication, regulatory sequence evolution, and the evolution of coding sequences, over evolutionary time. I will first examine what is known about the role of regulatory sequences and then discuss the contributions of coding sequences and gene duplication to the evolution of anatomy.

## Regulatory Sequences and the Evolution of Anatomy

Over the past decade or so, comparative studies of gene expression in diverse animals and plants, across all taxonomic levels, have revealed a general association between the gain, loss, or modification of morphological traits and changes in gene regulation during development [23,24]. Changes in the expression of an individual gene may evolve through alterations in cis-regulatory sequences or in the deployment and activity of the transcription factors that control gene expression, or both.

Progress toward elucidating the mechanisms governing the evolution of specific traits and genes has required the study of models in which genetic and molecular methods enable the identification and dissection of functional differences among populations or species. The traits and species for which such detailed analysis has been possible include the trichome [25–27], bristle [28], and pigmentation patterns [29] in fruit flies; flower coloration [30], architecture [31], and branch patterns [32] in plants; and limb [33] and axial diversity in vertebrates [34].

A handful of studies have genetically demonstrated that evolution at particular loci has affected the gain [32], loss [26,27,33], or modification of morphological traits [25]. These studies—highlighted below—have firmly eliminated coding sequences as a possible cause and thereby implicated regulatory sequence evolution at loci encoding pleiotropic transcription factors. In a few cases, direct evidence of functional changes in cis-regulatory elements has been obtained [34–36].

**Figure 2:**
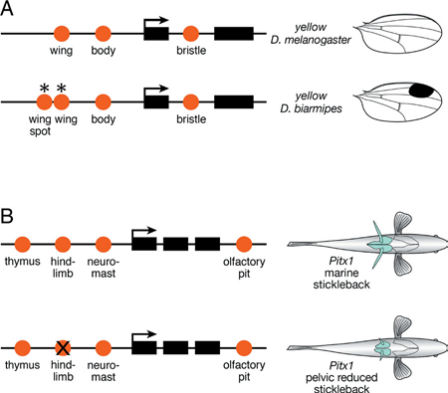
The Modular Architecture of the cis-Regulatory Regions of Pleiotropic Genes Enables the Independent Evolution of Gene Expression in Different Body Parts (A) Expression of the *yellow* pigmentation gene of Drosophila is controlled by several different cis-regulatory elements (red circles). Differences in the activity of selected elements (wing and wing spot) underlie differences in pigment patterns between species (Figure based on [35].) (B) Similarly, the expression of the *Pitx1* gene of vertebrates is inferred to be controlled by multiple elements (red circles). In pelvic-reduced stickleback fish, *Pitx1* expression is absent from the pelvic region. This is proposed to occur through of a selective loss of activity of the hindlimb regulatory element (cross through the red circle) (Figure based on [33].)

Fruit flies display all sorts of conspicuous patterns of black pigmentation on their head, thorax, abdomen, and wings. These patterns are regulated by a variety of well-conserved signaling pathways and transcription factors that control the spatial expression of the enzymes that promote or inhibit the formation of the pigment melanin [37]. In Drosophila melanogaster and other members of the genus, structural genes, such as *yellow*, are regulated by an array of *cis*-regulatory elements that govern their expression in different body parts, such as the wing and abdomen [36] and the bristles and larval mouthparts. This modular arrangement of *cis*-regulatory elements had suggested that gene expression and pigment patterns evolve independently in different body parts through changes in individual *cis*-regulatory elements. Recent studies have demonstrated this to be exactly the case [35,36] (Figure 2A).

There are several salient general features of the evolution of pigment patterns in fruit flies. Many or all of the structural genes involved are pleiotropic; they have roles in multiple parts of the body and in other physiological processes (for example, neurotransmitter synthesis and behavior). Furthermore, they are regulated, at least in part, by widely deployed, highly conserved pleiotropic regulatory proteins, some of which are themselves regulated by deeply conserved and evolutionarily stable global regulators of body pattern formation [29]. Thus, while the coding sequences of the structural and regulatory proteins are constrained by pleiotropy, modular *cis*-regulatory regions enable a great diversity of patterns to arise from alterations in regulatory circuits through the evolution of novel combinations of sites for regulatory proteins in cis-regulatory elements [35]. This diversity is produced by the sort of “tinkering” with existing components envisaged by Jacob [19].

Is what is true of coloration, true of more complex traits? It is possible that because body color patterns are so critical to organismal adaptation, the genetic systems that affect them might be more flexible than those governing more complex traits such as body organization, appendage formation, and other, more slowly evolving characters. The available evidence suggests, however, that the diversification of other traits that are governed by highly pleiotropic and well-conserved proteins can also be accounted for by regulatory sequence evolution.

For example, shifts in the rostrocaudal boundaries of *Hox* gene expression are associated with large-scale differences in axial patterning in vertebrates, arthropods, and annelids [24]. In one case, the *Hoxc8* gene of the chicken and mouse, differences in the function of one *cis*-regulatory element have been demonstrated to govern differences in gene expression boundaries in the neural tube and paraxial mesoderm [34].

While such differences in axial morphology are thought to evolve slowly and relatively infrequently, some features of the vertebrate skeleton, such as the pelvic skeleton of stickleback fish, evolve rapidly [38] and repeatedly [33]. Reduction of the pelvic fin, the homolog of the tetrapod hindlimb, is due to changes at the *Pitx1* locus [33]. The Pitx1 protein is a pleiotropic transcription factor that affects the development of multiple tissues in fish and mice, including the hindlimb. Pelvic-reduced sticklebacks have lost *Pitx1* expression in pelvic fin precursors, but possess a perfectly intact *Pitx1* coding region with no sequence changes relative to populations with fully formed pelvic structures. The only explanation consistent with these observations is that regulatory mutations in a *cis*-element governing expression in the pelvic fin precursors has selectively abolished *Pitx1* expression in this one part of the developing animal, while gene expression elsewhere is not affected (Figure 2B).

The crucial insight from the evolution of *Pitx1*, *yellow*, and *Hoxc8* is that regulatory mutations provide a mechanism for change in one trait while preserving the role of pleiotropic genes in other processes. This is perhaps the most important, most fundamental insight from evolutionary developmental biology. While functional mutations in a coding region are usually poorly tolerated and eliminated by purifying selection, even complete loss-of-function mutations in regulatory elements are possible because the compartmentation created by the modularity of cis-regulatory elements limits the effects of mutations to individual body parts.

Does this mean that coding sequences cannot contribute to morphological evolution? Not at all. There are several clear examples of functional sequence changes in proteins that affect form, and I will highlight them next. The key questions to keep in mind are, how often and under what circumstances do coding sequences of regulatory molecules functionally evolve?

## Coding Sequences and the Evolution of Anatomy

The body plans of arthropods and tetrapods have evolved around the use of a fairly stable complement of *Hox* genes in each phylum [24,39]. The stability of *Hox* gene number, and the conservation of *Hox* ortholog sequences and function, led to the initial impression that *Hox* proteins have not significantly diverged in function. However, it is now understood that several arthropod *Hox* proteins have changed in ways that are associated with shifts in form or developmental mechanisms, including the Hox3, Fushi tarazu, Ultrabithorax (Ubx), and Antennapedia [40] proteins. In the case of Hox3 and Fushi tarazu, Hox-type function has been lost in particular lineages while new functions have been gained. The Fushi tarazu protein of certain insects lost sequence motifs involved in Hox functions, and gained a motif for a new activity involved in segmentation [41,42]. Similarly, the Hox3 protein lost Hox function in insects and gained a novel dorsoventral axis patterning function. It subsequently underwent a duplication that produced two divergent genes involved in early patterning of the two body axes in one clade of flies [43–45].

In the Ubx protein, functional motifs evolved while the protein retained Hox function. Comparative and functional studies have shown that the carboxy terminus of the Ubx protein was extended in the crustacean lineage and serves as an activity-modulating domain [46]. In the insect lineage, this domain was replaced by a short glutamine/alanine-rich motif that has been well preserved throughout the course of more than 300 million years of insect evolution [47].

These arthropod Hox proteins demonstrate that some of the most conserved proteins can, under certain circumstances, evolve new and different activities. In these examples, selection against coding changes might have been relaxed because of functional redundancy among *Hox* paralogs. However, these events are, in the long span of the history of these lineages, rare relative to the extensive diversification of body forms. It must also be stressed that both *ftz* and *Hox3* (and its derivatives *zen* and *bcd*) acquired entirely novel regulatory elements that governed their expression in new domains and patterns. Furthermore, *Ubx* regulation has been extensively diversified among arthropods [24], including within the insects [48–50]. Thus, even in the infrequent instances of overt coding sequence evolution in regulatory proteins, regulatory sequence evolution is a critical component of functional evolution, and further diversification of gene function.

Are there more common and rapid means of evolving morphological diversity via coding mutations? Definitely. One prominent example is the melanocortin-1 receptor (MC1R), which modulates the quantity and type of melanin synthesis in melanocytes. Mutations in the *MC1R* gene are associated with scale, fur, or plumage color variation and divergence in a wide range of species [51]. The ecological significance of alternative phenotypes suggests that the *MC1R* gene has evolved under natural and sexual selection. The clear-cut case of *MC1R* evolution raises the question, why is coding sequence evolution so prevalent in the diversification of vertebrate pigmentation, while the evolution of gene regulation plays a central role in flower and fruit fly pigmentation?

There may be particular properties of MC1R that have enabled it to play this starring role. MC1R is a member of a family of five related receptors and is the only member involved in pigment synthesis regulation [52]. Thus, the structural and regulatory diversification of this receptor family (that is, the evolution of MC1R expression in melanocytes) has produced a protein that has a much greater degree of evolutionary freedom than more pleiotropic receptors. It should be noted that *MC1R* coding mutations result in body-wide effects on pigmentation, and do not create or alter spots, stripes, or other patterns. The evolution of spatial patterns of pigmentation in vertebrates is still likely to involve regulatory evolution in the expression of pigmentation proteins, or regulators of receptor activity [53], via mechanisms similar to those underlying the evolution of insect color patterns.

The widespread involvement of *MC1R* coding variation in the visible diversity of vertebrates may then be a relatively special case, enabled by the dedication of MC1R to pigmentation and its minimal pleiotropy. It would be expected that other, more pleiotropic proteins would be constrained in their sequence variation and, hence, their contribution to morphological variation. However, it has recently been shown that morphological variation in dog breeds is associated with variation in the length of repeated amino acid sequences in the coding regions of a variety of developmentally important transcription factors [54]. These repeats are encoded by microsatellite sequences that expand or contract at very high rates, and spontaneous or induced mutations of these sites affect visible traits. The extraordinary variation in repeat lengths, and their potential effects on morphology, raises the possibility that these repeats are a source of variation in natural populations. However, this variation may have accompanying deleterious, pleiotropic effects that, while manageable under domestication, would limit its contribution to evolution under natural selection.

## Gene Duplication and the Evolution of Anatomy

The history of *Hox* genes and the *MC1R* gene reflects that one condition contributing to the potential evolution of coding sequences is the generation of new genes by duplication. Ever since Ohno [14], and indeed well before [55], there has been widespread belief and expectation that gene duplication has been a major driving force in evolution. Empirical evidence suggests, however, that while gene duplication has contributed to the evolution of form, the frequency of duplication events is not at all sufficient to account for the continuous diversification of lineages. This conclusion is based primarily upon two sets of observations.

First, the estimated rate of gene duplication is about once per gene per 100 million years [56]. This figure suggests that gene duplication can contribute to genome evolution over longer spans of evolutionary time (for example, greater than 50 million years), but this rate is not sufficient to account for variation in populations (for example, quantitative trait differences) or for divergence among related species such as the 300,000 known species of beetles, or 10,000 species of birds.

Second, the relative infrequency of gene duplication is documented by the actual histories of key developmental regulatory gene families. For example, while it is very clear that during the early evolution of animals, there was an expansion in the number of *Hox* genes, and that during the early evolution of the vertebrates, there was an expansion in the number of *Hox* gene clusters, the number and diversity of *Hox* genes in highly diversified phyla, such as the arthropods and tetrapods, appears to have remained fairly stable for very long periods (perhaps approximately 500 million years). Other gene families, such as the Wnt family of signaling ligands, also exhibit deep ancestral complexity. Of 12 Wnt subfamilies known in vertebrates, 11 have been identified in a cnidarian [57]. Such deep ancestral complexity is much greater than would be expected under the hypothesis that diversity evolves primarily through the evolution of new genes [39,58]. Similarly, despite widespread speculation that the human genome would contain many more genes than other species, it does not, and the great majority of human genes have syntenic orthologs in the mouse [6].

Furthermore, the contribution of gene duplication to the evolution of form may be governed primarily by the divergence of the regulation of newly duplicated genes, rather than novel functions acquired by coding mutations. Both theoretical considerations and empirical data have suggested that the partitioning of the progenitor gene's functions may occur most often through regulatory mutations, or the partitioning of regulatory sequences in the original duplication event [59].

## The Relative Contribution of Regulatory and Coding Sequences to Anatomical Evolution

The examples I have described demonstrate that both regulatory sequences and coding regions of the genome can and do contribute to the evolution of form. The more subjective issue is whether, from the small sample of case studies mentioned here and in the literature, one can make (and defend) statements about the relative contribution of regulatory and coding sequence evolution to the evolution of anatomy. We are, after all, in much better position now to do so than King and Wilson were 30 years ago.

While the agnostic, “wait and see” position would appear safer, that would not at all be in keeping with the bold spirit of the pioneers who first wrestled with the question. Moreover, I argue that a trend is evident, and that that trend should, of course, inform ongoing and future work. Based upon (i) empirical studies of the evolution of traits and of gene regulation in development, (ii) the rate of gene duplication and the specific histories of important developmental gene families, (iii) the fact that regulatory proteins are the most slowly evolving of all classes of proteins, and (iv) theoretical considerations concerning the pleiotropy of mutations, I argue that there is adequate basis to conclude that the evolution of anatomy occurs primarily through changes in regulatory sequences.

This conclusion comes as no surprise, given the hypotheses of King and Wilson and others framed decades ago. Indeed, most aficionados of evolutionary developmental biology would find no news here. However, I am not convinced that what we have learned about the evolution of form is being adequately considered in comparative genomics and population genetics, where the potential role of regulatory sequence evolution appears to be a secondary consideration, or ignored altogether. This neglect has fundamental bearing on the issue that first drew King and Wilson's interest—the origins of differences between chimps and humans.

## Chimps and Humans Redux

The morphological differences between modern humans, human ancestors, and the great apes are the product of evolutionary changes in development. I have argued elsewhere [60] that the evolution of complex traits such as brain size, craniofacial morphology, cortical speech and language areas, hand and digit form, dentition, and body skeletal morphology must have a highly polygenic and largely regulatory basis. The great and difficult challenge, with the genome sequences of humans, chimps, and other mammals now available, is to map changes in genes to changes in traits. Many approaches are being taken, and a few intriguing associations of candidate genes and the evolution of particular traits have been discovered, such as the *FOXP2* gene and the evolution of speech [61], and the *MYH16* muscle-specific myosin pseudogene and the evolutionary reduction of the masticatory apparatus [62]. My concern here is not whether these specific associations did or did not play a role in human evolution; rather, my concern is the exclusive focus, by choice or by necessity, on the evolution of coding sequences in these and more genome-wide population genetic surveys of chimp–human differences [63].

There exists some disconnect between what studies in model species have underscored—the ability or sufficiency of regulatory sequences to account for the evolution of physical traits—and which models of evolution are implicitly or explicitly being tested when only coding sequence divergence is considered. Two stories concerning the *FOXP2* gene illustrate the dramatically different conclusions one might draw, depending upon the methodologies and assumptions applied.

The human *FOXP2* gene encodes a transcription factor, and mutations at the locus were discovered to be associated with a speech and language disorder [64]. The human *FOXP2* protein differs from the gorilla and chimp protein at just two residues, raising the possibility that the two replacements that occurred in the human lineage might be significant to the evolution of speech and language. Furthermore, population genetic analysis indicates that the *FOXP2* locus has undergone a selective sweep within the last 200,000 years of human evolution [61]. While it would certainly be convenient if the two changes in the FOXP2 protein were functional, the additional hypothesis must be considered that functional regulatory changes might have occurred at the *FOXP2* locus. In weighing alternative hypotheses of *FOXP2* or any gene's potential involvement in the evolution of form (or neural circuitry), we should ask the following questions. (i) Is the gene product used in multiple tissues? (ii) Are mutations in the coding sequence known or likely to be pleiotropic? (iii) Does the locus contain multiple *cis*-regulatory elements?

If the answers are yes to all of these questions, then regulatory sequence evolution is the more likely mode of evolution than coding sequence evolution. For *FOXP2*, this appears to be the case. *FOXP2* is expressed at multiple sites, not just in the brain, but in the lungs, heart, and gut as well [64,65]. Patients with the *FOXP2* mutation do have multiple neural deficits [66]. And, because *FOXP2* is expressed in different organs and different regions of the brain, it is certain to possess multiple regulatory elements. Furthermore, it is an enormous, complex locus, spanning some 267 kb. Based upon a simple average base pair divergence of 1.2%, there should be over 2,000 nucleotide differences between chimps and humans in this span. Because there is much more potential for functional divergence in non-coding sequences, there is no specific reason to favor coding sequence divergence over regulatory sequence divergence at *FOXP2*.

The discovery of *FOXP2* and its association with human speech has inspired consideration of the potential role of *FOXP2* in the evolution of vocalization in other animals, and here is where strikingly different conclusions were reached depending upon the hypothesis tested and the methodology used. Song learning has evolved in three orders of birds. There are some behavioral and neural similarities between bird song and human speech in terms of their being learned at critical periods and the involvement of auditory and motor centers and specialized brain centers. A standard comparative analysis of the *FOXP2* coding sequences of humans and song-learning and non-learning birds did not reveal any amino acid substitutions that were shared between song-learning birds and humans, nor any fixed differences between song-learning and non-learning birds. The study concluded there was “no evidence for its [*FOXP2*] role during the evolution of vocal learning in nonhuman animals” [67].

**Figure 3:**
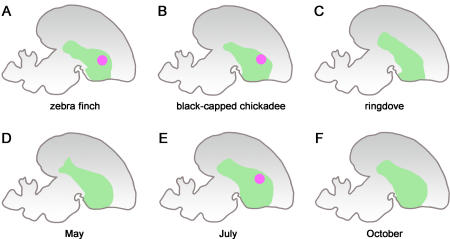
The Regulatory Evolution of FOXP2 and the Origins of Vocal Learning (A–F) The patterns of *FOXP2* expression in sections of bird brains are depicted The green area is the striatum. *FOXP2* is upregulated in the vocalization center known as Area X (pink spots) in vocal-learning species such as the zebra finch (A) and black-capped chickadee (B) but not in non-learning species such as the ringdove (C). (D–F) In the canary, *FOXP2* expression in Area X varies over seasons; elevated expression is associated with periods during which the song is plastic (pink spot). (Figure based on [68].)

In great contrast, when *FOXP2* mRNA and protein expression in the developing and adult brains of a variety of song-learners and non-learners were examined, a striking increase in *FOXP2* expression was observed in Area X, a center necessary for vocal learning that is absent from non-learners [68] (Figure 3A–3C). This increase occurs in zebra finches over the developmental period when vocal learning occurs. Furthermore, in adult canaries, seasonal changes in *FOXP2* expression were observed in Area X, associated with changes in the stability of the bird's song (Figure 3D–3F). Thus, remarkable changes in the regulation of *FOXP2*, but not the protein sequence, are correlated with the development and evolution of vocal learning in birds. These changes could arise through the evolution of FOXP2 *cis*-regulatory sequences, or of the regulatory or coding sequences of transcription factors that control FOXP2.

The contrast between the negative conclusions drawn from the analysis of coding sequences and the fascinating correlation revealed by the comparative study of gene regulation in vivo highlights the general inadequacies of, and potential error in, the exclusive analysis of coding regions when considering the evolution of anatomy. But that inadequacy applies more broadly than just to the evolution of form. While standard population genetic tests have been used to search human protein sequences for statistical evidence of positive selection [63,69], several examples of positive selection on cis-regulatory sequences of physiological genes are documented [70–72]. This includes the very clear case of the erythroid-specific loss of expression of the Duffy antigen chemokine receptor in populations resistant to Plasmodium vivax malaria [73]. This loss is due to a regulatory mutation that affects an erythroid *cis*-regulatory sequence but has no effect on receptor expression elsewhere in the body [74].

Any statements or claims, then, about the genetic changes that “make us human” must be weighed critically in light of the power and limitations of the methodology employed, and the scope of the hypotheses being tested. While it is understandable that some biologists have reached for the “low-hanging fruit” of coding sequence changes, the task of unraveling the regulatory puzzle is yet to come.

## Conclusion

The hypothesis of regulatory evolution put forward by King and Wilson 30 years ago was founded entirely on negative data, that is, the apparent insufficiency of coding sequence divergence to account for gross organismal differences. It has required several decades to obtain evidence that regulatory sequences are so often the basis for the evolution of form that, when considering the evolution of anatomy (including neural circuitry), regulatory sequence evolution should be the primary hypothesis considered. The analysis of regulatory sequence evolution poses particular challenges in that it is impossible to distinguish meaningless from functional changes by mere inspection. But, in nonhuman models where extensive experimental tools are available, there is cause for optimism that the contribution of regulatory sequences to evolution will be increasingly well understood in the near term. In order to approach the origins of human traits, much greater emphasis has to be placed on comparative studies of gene expression, regulation, and development in apes and other primates. This is precisely the requirement forecast by King and Wilson 30 years ago [1], only now we have the means to meet it.

## Abbreviations

MC1R: melanocortin-1 receptor
Ubx: Ultrabithorax
